# Modelling the factors associated with quality of life in women with osteoporosis: A cross-sectional study

**DOI:** 10.1016/j.gloepi.2024.100169

**Published:** 2024-10-09

**Authors:** Rahmatollah Moradzadeh, Maryam Zamanian, Maliheh Taheri

**Affiliations:** a Department of Epidemiology, School of Health. Arak University of Medical Sciences, Arak, Iran; b Department of Nursing, School of Nursing and Midwifery, Islamic Azad University-Sanandaj Branch, Sanandaj, Iran

**Keywords:** Quality of life, Osteoporosis, Structural equation model, Iran

## Abstract

**Background:**

Considering the important factors contributing to different health-related quality of life (HRQoL) subscales is essential for implementing preventive measures to increase the HRQoL among women with osteoporosis. We here evaluated the variables related to the mental and physical HRQoL in a sample of Iranian osteoporotic women.

**Methods:**

In this cross-sectional study, the participants included 111 women with osteoporosis in 2013. Physical and mental of HRQoL were measured by the ECOS-16 questionnaire. Other variables included BMD t-score (Osteoporosis was diagnosed based on bone mineral density (BMD) with BMD t-score < −2.5), age, body mass index, educational level, marital status, duration of the disease, history of bone fracture in the past year, the number of pregnancies, and weekly walking hours. Final regression coefficients were obtained based on the total effects of estimations (decompositions of effects into direct, indirect and total effects) by structural equation model (SEM) analysis.

**Results:**

The mean scores of physical and mental domains of HRQoL were 54(21) and 54(25), respectively. The mean of BMD t-score was −3.2 (0.9). Based on the regression coefficients obtained in the SEM model, weekly walking hours(2.2), number of pregnancies (−1.2), and history of bone fracture in past year(−2.9) were the more important determinants of physical aspect of HRQoL than other included variables of this study. Furthermore, age over than 60 (−9.1), history of bone fracture in past year(−4.8), weekly walking hours(2.3), marital status(−5), and education level (3.9)influenced the mental aspect of HRQoL.

**Conclusions:**

Social and life style factors tend to impact on physical and mental domains of HRQoL, a measure that is influenced by multiple factors among postmenopausal women. In this respect, these obtained factors should be considered for health planning to improve the physical and mental domains of HRQoL among postmenopausal women.

## Introduction

Population aging is occurring rapidly worldwide [1]. The bone mineral density (BMD) declines with age, especially in postmenopausal women [2]. According to the world health organization (WHO), the prevalence of osteoporosis is about 30 % among postmenopausal women [3]. Generally, losing bone begins at a younger age and progresses at a faster rate in women compared to men [4]. In fact, osteoporosis is four times more prevalent in women than men [4]. The prevalence of osteoporosis among women around the world is 23.1 %. Furthermore, highest prevalence of osteoporosis is among African women (prevalence: 42.4 %), while it is 24.3 % in Asia [1].

Osteoporosis disposes patients to bone fractures imposing remarkable medical, social, and economic burden and jeopardizing the patients' quality of life (QoL) [3,5]. Quality of life is a measure of multi-dimensional concepts that goes beyond the biological function of a person and shows the overall well-being of a person from different aspects of life [6]. The health-related quality of life (HRQoL) is a subgroup of QoL assessing physical and emotional features. Indeed, the HRQoL is the objective assessment of subjective feelings. In epidemiologic studies, HRQoL is used to estimate the burden and morbidity of diseases, as well as to optimize health care applications and improve the cost-effectiveness of therapeutic interventions [7]. Some studies have estimated that HRQoL of osteoporotic women was lower than that of normal population [8]. Different factors contributing to the HRQoL of osteoporotic women include age, walking for exercise, cerebrovascular disease, osteoarthritis, hypertension, higher perceived stress, poor glycemic control, waist circumference, sitting time per day and as well as a longer postmenopausal period [9]. There are valid and reliable scales or tools to measure HRQOL of different population or just are applicable for disease-specific HRQOL. some of them are generic tools for example EuroQol 5-dimension questionnaire (EQ-5D), 36-Item Short Form Survey (SF-36), the Sickness Impact Profile (SIP) and Nottingham Health Profile (NHP) that are used for osteoorosis patients, but some others, are osteoporosis-specific including the Osteoporosis Quality of Life Questionnaire (OQLQ), Osteoporosis Assessment Questionnaire (OPAQ-SV) and the The Quality of Life Questionnaire (QUALEFFO) [10,11]. Furthermore, Short Osteoporosis Quality of Life Questionnaire (ECOS-16), in principal, provides a promising alternative to longer generic and disease-specific questionnaires, particularly for use in clinical practice, although perhaps also in clinical research. [12,13].

Studying the important factors contributing to different HRQoL subscales is essential for implementing preventive measures to increase the HRQoL among osteoporotic patients. Sanandaj is one of the locations that prevalence of osteoporosis is high (34.4 % among postmenopausal women) and indeed prevalence of the osteoporosis-related risk factors is high [14]. Therefore, we here evaluated the variables related with the mental and physical HRQoL by ECOS-16 in a sample population of Iranian osteoporotic women.

## Methods

### Study participants

In this cross-sectional study, the participants included 111 women with osteoporosis diagnosed by a rheumatologist in a rheumatology clinic in Sanandaj, Iran in 2013. The convenience sampling was applied for recruitment of the participants. In other words, among the clients of the rheumatologist's office, people with osteoporosis were invited to participate in the study, and if they agreed to participate in the study, a questionnaire and data related to their disease status were recorded and collected. For reliable correlation and regression analyses and in according to the Cohen [15] suggestion, the sample size was decided as 110 to achieve the power of 0.80, the medium effect size of 0.15, and an alpha level of 0.05 [16].

Guidelines related to osteoporosis recommended that routine bone mineral density (BMD) screening should done by use of dual-energy x-ray absorptiometry (DXA) scans [17]. A BMD at the femoral neck equal to or less than 2.5 standard deviations below the mean for a young person of the same sex is diagnostic of osteoporosis. This is reported as a t-score of −2.5 or less [18]. Therefore, osteoporosis was diagnosed based on BMD with t-score < −2.5 [19,20]. The questionnaires were completed either self-administratively (for educated patients) or by face-to-face interviews (for illiterate patients). The interviews were conducted by a nurse educated in osteoporosis and blinded to the study aims. The interviews were conducted in a private room to make the respondents feel comfortable.

The ethical issues in medical research were completely considered, and the study was approved by the ethic committee. All the participants completed an informed consent. Furthermore, all the completed questionnaires were kept confidential by the researchers.

### The variables

The two main outcome variables in this study included physical and mental functions of HRQoL. These variables were measured by the ECOS-16 questionnaire which has been cross-cultural adapted in Persian language in a previous study in Sanandaj by Moradzadeh et al. [12] Available in Supplementary), reliability based on Cronbach's alpha for the Persian ECOS-16 questionnaire was 0.84, and ECOS-16 was valid and reliable instrument among Persian population [12]. Each item was scored between 1 and 5 with higher scores indicating a better HRQoL. In this study, the obtained scores were multiplied by 100. The 16 items of the ECOS-16 questionnaire were categorized into two main domains of physical (pain and physical subscales) and mental (fear of illness and psychological function) HRQoL. Other variables included BMD t-score, age, body mass index (BMI), educational level, marital status, duration of the disease (duration from diagnosis to the time of interview), history of bone fracture in the past year, the number of pregnancies, and weekly walking hours.

### Statistical analysis

Statistical analyses were carried out in Stata 12.0 software. Following the descriptive analysis, statistically significant differences between HRQoL dimensions and each of categorical variables were assessed by independent samples student *t*-test or analysis of variance (ANOVA) and bonferroni's test were used for multiple comparisons. Spearman correlation coefficient was applied to determine the correlation between quantitative variables. The little missing values (e.g. 10 and 2 data points in BMI and Educational level, respectively) were imputed using expectation-maximization (EM) algorithm. Using an iterative approach, the means, covariance matrixes, and the correlations between the quantitative variables were investigated. Nevertheless, the covariance-based statistics calculated by EM algorithm using the imputed values may underestimate their respective values [21]. Stata's “mi” command computes an EM covariance matrix as part of the imputation process [22]. The Little's test, indicating that the evidence against missing completely at random was not strong (*p*-value = 0.9).

The SEM analysis was used as the path model. The linear regression models in SEM fit into a single-level data. However, the SEM is not merely a linear regression but also a way of thinking, writing, and estimating parameters. The final regression coefficients were obtained based on the total effects of estimates (i.e. decompositions of effects into direct, indirect and total effects). The goodness of fit model was evaluated by Chi Square test and degrees of freedom (df), the root mean square error of approximation (RMSEA), probability RMSEA ≤ 0.05, and comparative fit index (CFI), and Tucker–Lewis index (TLI) indices. Furthermore, CFI > 0.95 TLI > 0.90 is used as the criterion of acceptable fit [23]. Other indices that included were as GFI, IFI, NFI and SRMR. The major assumptions associated with SEM include were considered, including multivariate normality, no systematic missing data, sufficiently large sample size, and correct model specification [24]. SEM analysis was applied in Stata 17.0.

## Results

### Demographic characteristics

The demographic characteristics of study participants have been presented in Table 1. The most of the participants were married and illiterate. Seventeen percent of the included patients had history of bone fractures in the past year. The mean age of the studied sample population was 59(10) years old. Furthermore, the average BMI was 28 kg/m^2^. The mean number of pregnancies was 5. The means of weekly walking hours and disease duration were 1.4 h and 6 years respectively.

**Table 1:** The differences among characteristics versus quality of life (*n* = 111).

Variable Name			Physical quality of life		Mental quality of life	
		n(%) Mean (SD)	Mean	SD	Mean	SD
Marital status						
	married	96(86)	54	20	55	25
	Single/ Divorced /widowed	15(14)	54	26	50	23
Education level						
	illiterate	58(52)	46	20	47	26
	Elementary/middle school	29(26)	61	20	57	23
	High school and more	24(22)	65	19	68	21
History of bone fracture in the past year						
	No	92(83)	55	21	56	25
	Yes	19(17)	50	23	48	24
Age						
	≤ 60	63(57)	58	20	61	24
	>60	48(43)	49	21	45	24

Considering different subscales of HRQoL, the mean scores of pain, physical function, psychological function and fear of illness were 49(24), 59(22), 70(23) and 39 (37), respectively. Furthermore, the mean scores of physical and mental domains of HRQoL were 54 (21) and 54 (25) respectively. Finally, the mean of BMD t-score was −3.2(0.9).

As shown in Table 1, the mean scores of physical and mental domains of HRQoL differed by 9 and 16 points by age older and younger than 60 years and by 19 and 21 points between illiterate and high school graduates, respectively.

However, there were a difference mean score on physical and mental domains of HRQoL 0 and 5 points between married and single/divorced/widowed groups and 5 and 8 points between with and without history of bone fracture during the past year, respectively.

**Fig. 1:**
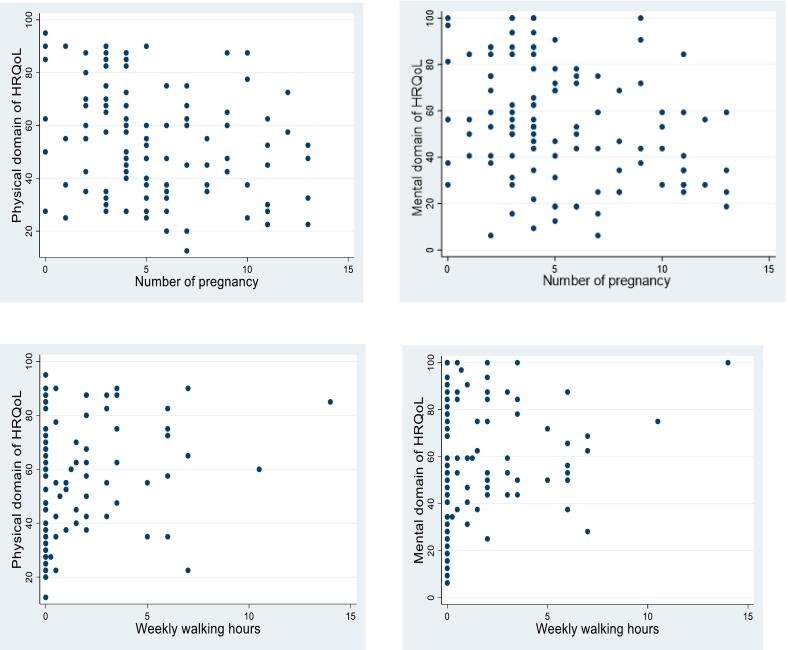
Scotter plots of physical and mental domains of HRQoL and number of pregnancy and weekly walking hours.

There was a moderate correlation between physical and mental aspects of HRQoL (*r* = 0.6, CI 95 %: 0.4, 0.7). There were also negative and little correlations for the mean scores of physical and mental HRQoL with the number of pregnancies, weekly walking hours, BMI and duration of disease (Table 2 and Fig. 1).

**Table 2:** Correlations among subject characteristics, and physical and mental quality of life, (n = 111).

Variables	Mean (SD)	Physical quality of life	Mental quality of life
		r⁎ (CI 95 %)	r⁎ (CI 95 %)
BMI	28 (5)	−0.2 (−0.3, 0.1)	−0.1 (−0.3, 0.1)
Number of pregnancies	5 (3)	−0.3 (−0.4, −0.1)	−0.3 (−0.4, −0.1)
Weekly walking hours	1 (2)	0.3 (0.1, 0.5)	0.3 (0.2, 0.5)
Duration of the disease	6 (6)	−0.2 (−0.4, 0.1)	−0.2 (−0.3, 0.1)

⁎ r: Spearman Correlation Coefficient.

### SEM analysis

**Fig. 2:**
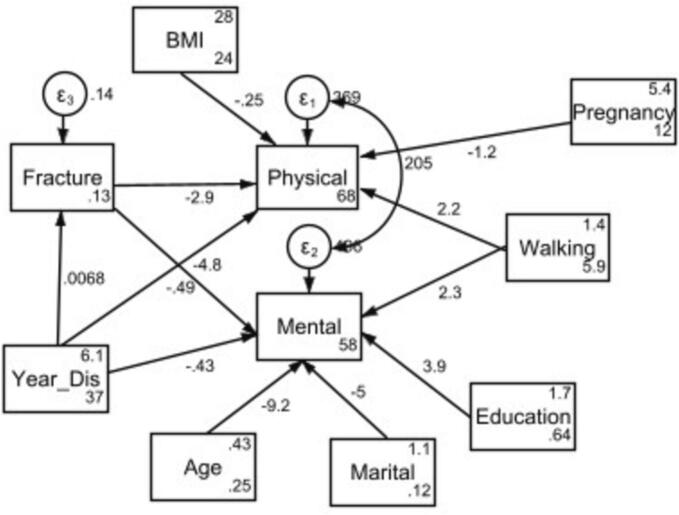
path models with regression coefficients on physical and mental quality of life in women with osteoporosis. (BMI: Body Mass Index; Physical: Physical quality of life; Mental: Mental quality of life; Walking: Weekly walking hours; Pregnancy: Number of pregnancies; Education: Education levels; Marital: Marital status; Year_Dis: Duration of the disease; Fracture: history of bone fracture in the past year).

The path diagram achieved by SEM analysis has been displayed in Fig. 2. The associations between the independent variables (i.e. age, BMI, educational level, marital status, duration of disease, history of bone fracture in the past year, the number of pregnancies, and weekly walking hours) and dependent variables (i.e. physical and mental HRQoL aspects) were determined to develop a fit model. In addition, the history of bone fracture in the past year was considered as the mediator between disease duration and physical and mental HRQoL dimensions. A mediator is an intermediate variable between an exposure and the outcome, which is influenced by the exposure on the causal pathway to the outcome [25]. As the disease duration increases, patients are exposed to more bone fractures and this issue may change their physical and mental HRQoL dimensions.

The goodness of fit indices indicated suitable fitness model on our data. Chi Square test and related degree of freedom were obtained 11.7 and 11 respectively (*P*-value = 0.4). The RMSEA and probability RMSEA ≤ 0.05 were calculated as 0.02 and 0.6 respectively. In addition, the CFI and TLI indices were obtained as 0.99 and 0.98, respectively. Other indices values including GFI, IFI, NFI and SRMR were 0.8, 0.99, 0.9 and 0.033, respectively.

The regression coefficients calculated based on the SEM model have been shown in Table 3. In this regard, more pregnancies were associated with decreased physical HRQoL(regression coefficient: −1.2). Furthermore, higher weekly walking hours predicted higher physical HRQoL(regression coefficient: 2.2). The duration of disease was negatively associated with physical HRQoL(regression coefficient: −0.5). Moreover, decreasing of physical score of HRQoL were associated with having history of bone fracture in the past year (regression coefficient: −2.9) and increasing of BMI (regression coefficient: −0.3).

**Table 3:** Total effects of the variables on the sub scales of quality of life based on the SEM analysis.

Subscales of quality of life	Variables	coefficient	95 % Confidence Intervals
Physical quality of life	History of bone fracture in the past year	−2.9	(−12.5, 6.7)
	BMI	−0.3	(−0.9, 0.4)
	Number of pregnancies	−1.2	(−2.2, −0.2)
	Weekly walking hours	2.2	(0.7, 3.7)
	Duration of the disease	−0.5	(−1.1, 0.1)
Mental quality of life	History of bone fracture in the past year	−4.8	(−15.9, 6.4)
	Age higher than 60 years	−9.1	(−17.2, −1.1)
	Weekly walking hours	2.3	(0.4, 4.1)
	Marital status	−5.0	(−16.3, 6.3)
	Education level	3.9	(−1.3, 9.1)
	Duration of the disease	−0.5	(−1.2, 0.2)

Goodness of fit indices: Chi Square = 11.7, DF = 11 (*P*-value = 0.4), RMSEA = 0.023, Probability RMSEA ≤ 0.05 = 0.62, CFI = 0.99, TLI = 0.98 and GFI, IFI, NFI and SRMR were 0.8, 0.99, 0.9 and 0.033, respectively.

Furthermore, individuals older than 60 years old resulted in poorer mental HRQoL (regression coefficient: −9.1) while longer weekly walking hours predicted better mental HRQoL (regression coefficient: 2.3). Moreover, decreasing of mental score of HRQoL were associated with having history bone fracture in the past year (regression coefficient: −4.8), higher duration of the disease (regression coefficient: −0.5), lower education level (regression coefficient: 3.9) and marital status with single/divorced/widowed (regression coefficient: −5.0).

## Discussion

### Core summary findings

Our findings highlighted that having a history of bone fracture in the past year, increasing of number of pregnancies and low weekly walking hours were associated with low physical HRQoL. On the other hand, having a history of bone fracture in the past year, age, weekly walking hours, marital status, education level influenced mental HRQoL in our patients.

Comparisons with Existing Literature.

In other studies, patients' awareness of reduced BMD has been highlighted as a factor intensifying anxiety, as well as fear of falling and bone fractures in osteoporotic patients which in turn results in reduced independence and poorer HRQoL [26].

In present study, mental HRQoL reduced with increasing age which is in parallel with previous studies. The physical functioning of older women has also been limited due to the fear of falling and possible fractures [27,28].

Weekly walking hours has been associated with longer life span, lower risk of diseases and more desirable HRQoL in osteoporotic patients [29]. In line with other studies [28,30], we also found that weekly walking hours had a positive impact on both physical and mental HRQoL of osteoporotic women.

The number of pregnancies negatively associated with the physical HRQoL of osteoporotic women in our study which is consistent with a previous report noting that women with more pregnancies had lower HRQoL [31]. Women's health is substantially decreased during and after pregnancy. In fact, maternal BMD decreases by about 3 % during each pregnancy [32]. After delivery, other activities and problems including breastfeeding, child-rearing, limited physical activity due to obesity, postpartum stress, low back pain, pelvic pain, and other issues in parous woman exaggerate the reduced maternal health leading to poorer HRQoL [31].

A negative association has been reported between BMI and HRQoL in some studies. Actually, interventions for weight loss improved HRQoL over time [[33], [34], [35]]. In the present study, BMI negatively correlated with physical and mental HRQoL.

Women with higher education have probably more knowledge and better understanding of their disease which make them to adhere to their medical regimen more effectively. Furthermore, educated women are expected to have higher coping capabilities with the condition [36]. Accordingly, higher education has been noted as a mitigating factor toward HRQoL in multiple studies [28,37,38].

In our study, higher BMI, history of fractures in the past year and being single were associated with lower scores of physical and mental HRQoL.

### Limitations

One of the limitations in our study included self-reported data collection. Although self-reporting is a routine approach in medical research [39], there may be potential risk of misclassification bias on the findings [40] which can be decreased by applying bias analysis methods [39,41]. On the other hand, selection bias is another committed bias. The included patients were recruited from a rheumatology clinic; however, some patients might refer to other specialists such as internists, or orthopedist. So, we didn't have access to some patients that might lead to selection bias and generalizability of findings should be avoided. Furthermore, there were a number of other variables that we could not assess them, and it is suggested to include these in future studies. Also, the impact of the mediators and interactions between the variables should be considered deeper.

Implications for Policy, Practice and Future Research Direction.

It is recommended that physicians pay attention to the HRQoL using the ECOS-16 questionnaire in the clinical field. Regarding the variables obtained in this research, physicians can prescribe to their patients to take a walk during the week, especially in people with a history of bone fractures in the past year, and with a high number of pregnancies to improve the physical quality of life.

Based on the key findings obtained in this study, in order to improve the mental quality of life among osteoporosis patients, it is emphasized that in the clinical field, especially in women over 60 years old, people with a history of bone fracture in the last year and patients with single/divorced/widowed marriage status and low education levels should emphasize and pay special attention to preventive measures, including increasing physical activity and weekly walks.

Furthermore, in future research, it is recommended to study other important variables such as the interval between menstruation and menopause, the use of contraceptives, more details about physical activity and sitting time, occupation and sun exposure.

## Conclusions

Social and life style factors tend to impact on physical and mental domains of HRQoL, a measure that is influenced by multiple factors among postmenopausal women. Some factors have a negative association on physical domain of HRQoL. Based on this SEM analysis, these factors include the history of bone fracture in past year, BMI, number of pregnancies and duration of the osteoporosis. While, weekly walking hours have a positive association.

The negative related factors with mental domain of HRQoL include history of bone fracture in past year, age over than 60, single, divorced, widowed marriage status, high duration of osteoporosis. Other factors have a positive association on the mental domain of HRQoL, such as the educational level of the postmenopausal women and weekly walking hours. Thus, appropriate interventions need to be made by health policymakers that aim to promote physical and mental domains of HRQoL among these women. In this respect, these obtained factors should be considered for health planning to improve the physical and mental domains of HRQoL among postmenopausal women.

## Ethics approval and consent to participate

The study was approved by the Ethic committee of Arak University of Medical Sciences, Arak, Iran (ethic code: IR.ARAKMU.REC.1394.382) and written informed consent was obtained from all participants. The study was conducted according to the guidelines of the Declaration of Helsinki. All authors confirm that all experiments were performed in accordance with relevant guidelines and regulations.

## Consent for publication

Not applicable.

## Funding

Not applicable.

## CRediT authorship contribution statement

**Rahmatollah Moradzadeh:** Writing – original draft, Visualization, Validation, Supervision, Software, Resources, Project administration, Methodology, Investigation, Funding acquisition, Formal analysis, Data curation, Conceptualization. **Maryam Zamanian:** Writing – review & editing, Software, Methodology, Investigation, Formal analysis, Data curation. **Maliheh Taheri:** Writing – review & editing, Software, Methodology, Investigation, Formal analysis, Data curation.

## Declaration of competing interest

The authors declare that they have no known competing financial interests or personal relationships that could have appeared to influence the work reported in this paper.

## Data Availability

The datasets generated and/or analysed during the current study are not publicly available due the other manuscripts are ongoing publishing, but are available from the corresponding author on reasonable request.

## Appendix A Supplementary data

Supplementary material - Persian Questionnare. Image 1
