# Supplementary material for: Modelling the factors associated with quality of life in women with osteoporosis: A cross-sectional study
Source: Glob Epidemiol. 2024 Oct 9;8:100169. doi: 10.1016/j.gloepi.2024.100169 (PMC11550349; doi:10.1016/j.gloepi.2024.100169)
Supplement: Supplementary file 1 — Supplementary material - Persian Questionnare. [file mmc1.docx]

**پرسشنامه**

سن

شغل

تعداد سالهای ابتلا

وضعیت تأهل

سطح تحصیلات

سابقه هم ابتلایی(Comorbidity)

سابقه ابتلا در فامیلهای درجه یک

سابقه تعداد شکستگی

وزن

قد

BMI (شاخص توده بدنی)

سن شروع یائسگی

وجود شکستگی های مهره ای و غیر مهره ای و محل

مقدار تراکم توده استخوانی

**در طول هفته گذشته و به دليل مشكلات كمري ناشي از پوكي استخوان به سوالات زير پاسخ دهيد:**

1. در هفته گذشته چند بار كمردرد داشته ايد؟

الف) كمردرد نداشته ام

ب) 1 روز

ج) 3-2 روز

د) 6-4 روز

ه) هر روز

1. **شدت كمردرد تان چگونه بوده است؟**

الف) كمردرد نداشته ام

ب) خفيف

ج) متوسط

د) شديد

ه) غير قابل تحمل

1. **زجر(distress) يا ناراحتي(discomfort ) شما به دليل درد در ايستادن طولاني چگونه بوده است؟**

الف) نداشته ام

ب) خفيف بوده است

ج) متوسط بوده است

د) شديد بوده است

ه) خيلي شديد بوده است

1. **زجر يا ناراحتي شما ناشي از درد در خم و راست شدن چگونه بوده است؟**

الف) نداشته ام

ب) خفيف بوده است

ج) متوسط بوده است

د) شديد بوده است

ه) خيلي شديد بوده است

1. **آيا وضعيت خواب شما به دليل كمردرد در طي هفته گذشته مختل شده است؟**

الف) اصلا

ب) يك شب

ج) دو شب

د) سه يا چهار شب

ه) هر شب

1. **در انجام كارهاي خانه ( يا فعاليتهاي روزمره ) چه قدر با مشكل مواجه شده ايد:**

الف) مشكلي نداشته ام

ب) مشكل خفيفي داشته ام

ج) مشكل متوسطي داشته ام

د) مشكل زيادي داشته ام

ه) نتوانسته ام كاري انجام دهم

1. **توانايي شما در بالا رفتن از پله ها چگونه بوده است؟**

الف) بدون هيچ مشكلي

ب) با مشكل كمي

ج) با حداقل يك بار استراحت مي توانم بالا بروم

د) فقط با كمك مي توانم بالا بروم

ه) نمي توانم از پله ها بالا بروم

1. **آيا در پوشيدن لباس هايتان مشكلي داريد؟**

الف) مشكلي ندارم

ب) با مشكل كمي مي توانم لباس هايم را خودم بپوشم

ج) با مشكل در حد متوسطي مي توانم لباس هايم را خودم بپوشم

د) بعضي وقتها در پوشيدن لباسم نياز به كمك دارم

ه) بدون كمك نمي توانم لباس هايم را بپوشم

1. **در خم و راست شدن چقدر مشكل داريد؟**

الف) مشكلي ندارم

ب) مشكل خفيفي دارم

ج) مشكل متوسطي دارم

د) مشكل زيادي دارم

ه) نمي توانم خم و راست شوم

1. **چقدر پياده روي تان محدود شده است؟**

الف) محدود نشده است

ب) محدوديت خفيفي دارم

ج) محدوديت متوسطي دارم

د) خيلي محدود شده ام

ه) نمي توانم پياده روي كنم

1. **ملاقات با دوستان يا خويشاوندانتان چقدر مشكل شده است؟**

الف) مشكلي ندارم

ب) مشكل خفيفي دارم

ج) مشكل متوسطي دارم

د) مشكل زيادي دارم

ه) نمي توانم به ملاقات آنها بروم

1. **چقدر احساس افسردگي (downhearted) داريد؟**

الف) اصلا

ب) به ندرت

ج) بعضي اوقات

د) غالبا

ه) هميشه

1. **آيا اميدوار به آينده هستيد؟**

الف) هميشه

ب) غالبا

ج) بعضي اوقات

د) به ندرت

ه) اصلا

1. **آيا احساس بيچارگي(frustrated ) داريد؟**

الف) اصلا

ب) به ندرت

ج) بعضي اوقات

د) غالبا

ه) هميشه

1. **آيا از اينكه بيفتيد(falling) مي ترسيد؟**

الف) اصلا

ب) به ندرت

ج) بعضي اوقات

د) غالبا

ه) هميشه

1. **آيا از شكسته شدن اعضاي بدنتان مي ترسيد؟**

الف) اصلا

ب) به ندرت

ج) بعضي اوقات

د) غالبا

ه) هميشه
